# (1*S*,3*S*)-Methyl 2-benzyl-6,7-dimeth­oxy-1-phenyl-1,2,3,4-tetra­hydro­isoquinoline-3-carboxyl­ate

**DOI:** 10.1107/S1600536811017430

**Published:** 2011-05-14

**Authors:** Tricia Naicker, Thavendran Govender, Hendrik. G. Kruger, Glenn. E. M. Maguire

**Affiliations:** a School of Pharmacy and Pharmacology, University of KwaZulu–Natal, Durban 4000, South Africa; b School of Chemistry, University of KwaZulu–Natal, Durban 4000, South Africa

## Abstract

In the title compound, C_26_H_27_NO_4_, the heterocyclic ring assumes a half-chair conformation and inter­molecular C—H⋯O inter­actions help to construct the three-dimensional network within the crystal packing.

## Related literature

The title compound is a precursor to chiral catalysts bearing a tetra­hydro­isoquinoline (TIQ) backbone. TIQ catalyst precursors have shown to be efficient for several asymmetric transformations, see: Chakka *et al.* (2010 ▶); Kawthekar *et al.* (2010 ▶). For related structures, see: Naicker *et al.* (2009 ▶, 2010 ▶, 2011 ▶). For the assignment of the absolute stereochemisty by NMR, see: Aubry *et al*. (2006 ▶).
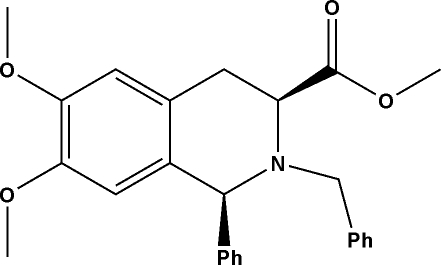


## Experimental

### 

#### Crystal data

C_26_H_27_NO_4_
*M* *_r_* = 417.49
Monoclinic, 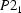

*a* = 9.7797 (7) Å
*b* = 5.4646 (4) Å
*c* = 20.6959 (15) Å
β = 96.986 (1)°
*V* = 1097.82 (14) Å^3^
*Z* = 2
Mo *K*α radiation
μ = 0.09 mm^−1^
*T* = 173 K
0.85 × 0.07 × 0.06 mm

#### Data collection

Bruker Kappa DUO APEXII diffractometer
Absorption correction: multi-scan (*SADABS*; Sheldrick, 2008*a* ▶) *T*_min_ = 0.931, *T*_max_ = 0.995
20624 measured reflections
3032 independent reflections
2764 reflections with *I* > 2σ(*I*)
*R*_int_ = 0.030

#### Refinement

*R*[*F* ^2^ > 2σ(*F* ^2^)] = 0.031
*wR*(*F* ^2^) = 0.087
*S* = 1.05
3032 reflections
280 parameters
1 restraint
H-atom parameters constrained
Δρ_max_ = 0.21 e Å^−3^
Δρ_min_ = −0.19 e Å^−3^

### 

Data collection: *COLLECT* (Nonius, 2000 ▶); cell refinement: *DENZO-SMN* (Otwinowski & Minor, 1997 ▶); data reduction: *DENZO-SMN*; program(s) used to solve structure: *SHELXS97* (Sheldrick, 2008*b* ▶); program(s) used to refine structure: *SHELXL97* (Sheldrick, 2008*b* ▶); molecular graphics: *OLEX2* (Dolomanov *et al.*, 2009) ▶; software used to prepare material for publication: *SHELXL97*.

## Supplementary Material

Crystal structure: contains datablocks I, global. DOI: 10.1107/S1600536811017430/gw2101sup1.cif

Structure factors: contains datablocks I. DOI: 10.1107/S1600536811017430/gw2101Isup2.hkl

Additional supplementary materials:  crystallographic information; 3D view; checkCIF report

## Figures and Tables

**Table 1:** Hydrogen-bond geometry (Å, °)

*D*—H⋯*A*	*D*—H	H⋯*A*	*D*⋯*A*	*D*—H⋯*A*
C18—H18⋯O3^i^	0.95	2.51	3.445 (2)	168
C25—H25*A*⋯O1^ii^	0.98	2.43	3.183 (2)	133

Symmetry codes: (i) 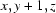; (ii) 
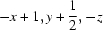
.

**Fig. 1.**
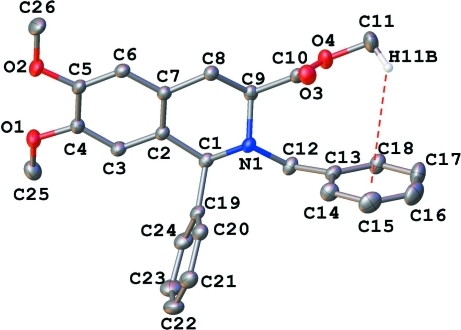
The molecular structure of the title compound with the atom numbering scheme and the intramolecular C11—H11B···π interaction. Displacement ellipsoids are drawn at the 40% probability level. Hydrogen atoms have been omitted for clarity.

**Fig. 2.**
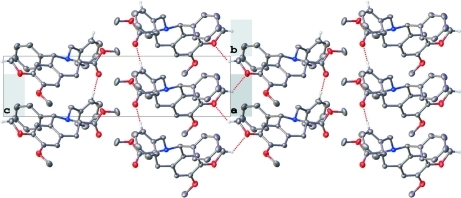
A partial projection of the title compound, viewed along the [100] plane.

## supplementary crystallographic information

### Comment

Chiral catalysts containing a tetrahydroisoquinoline (TIQ) backbone have proven to be very successful in our research group. These TIQ catalyst precursors have shown to be efficient for several asymmetric transformations. (Chakka *et al.*, 2010, Kawthekar *et al.*,2010 and Naicker *et al.*, 2010) The title compound (Fig. 1) is a precursor in the synthesis of several novel chiral ligands containing the TIQ framework.

The absolute stereochemistry of the crystal was confirmed to be S,*S* at C1 and C9 positions respectively by proton NMR spectroscopy. We recently reported the crystal structure of the *R*,*S* diastereomer at the C1 and C9 positions respectively. (Naicker *et al.*, 2009)

Interestingly, there are several significant differences between these diasteromeric crystals. The title compound crystallizes with monoclinic (P21) symmetry while its diastereomer has triclinic (P1)symmetry. Also the *N*-containing six membered ring assumes a half chair conformation [Q=0.5312 (16) Å, θ= 53.39 (17)° and φ=324.7 (2)°] as apposed to a half boat conformation (Fig. 1). This heterocyclic ring shape affects the position of the ester moiety relative to the phenyl ring at the C1 position. The torsion angle for C1—N1—C9—C10 is -171.7 (1)° while for the diastereomer this angle was 66.0 (2)°. In addition, the *N*-benzyl and phenyl ring at C1 exisit in a *cis* orientation along the N1—C9 bond with a dihedral angle of 70.2 (2)° while for the diasteromer they are *trans* to each other with a dihedral angle of -64.7 (1)°. From the plain formed by the atoms C1—C2—C7—C8—N1—C9 the maximum displacement from planarity for N1 is 0.334 Å and for C9 0.360 Å.

A single intramolecular interaction between H11B and the phenyl ring attached to C12 (2.862 Å) is evident (Fig. 1). Two specific intermolecular short contacts originating from methoxy O1 and the ester O3 to different C–H groups (Fig. 2) link the molecules together in the crystal (Table 1). This arrangement results in chains parallel to the *a* axis. In the chain, the molecules are arranged so that their tails, linked by these C—H···O interactions, protrude to the outer edges of the chain, and their heads point towards the core of the chain.

### Experimental

To a solution of (1*S*,3*S*)-methyl 6,7-dimethoxy-1-phenyl-1,2,3,4-tetrahydroisoquinoline-3-carboxylate (500 mg, 1.52 mmol) in acetonitrile (20 ml), solid K_2_CO_3_ (635 mg, 4.58 mmol) was added followed by benzyl bromide (286 mg, 1.67 mmol) at ambient temperature. There after the reaction mixture was refluxed for 3 h. Completion of the reaction was monitored with TLC using hexane/ethyl acetate (60:40, *R*_f_ =0.5). The solvent was evaporated and 30 ml of ethylacetate was added, washed with 2 × 10 ml of water, the organic layer was separated, and dried over anhydrous MgSO_4_. The solvent was evaporated under reduced pressure to afford crude product, which was purified by column chromatography using hexane:ethyl acetate (60:40) as the eluent to yield pure product. (0.44 g, 90%) as a white solid.

Melting point: 420 K. [α]^20^_D_ +3.03 (c 0.1 in CHCl_3_).

IR (neat): 2925, 1729, 1513, 1216, 747 cm^-1^.

^1^H NMR (400 MHz, CDCl_3_) δ 7.37 (d, *J* = 7.7 Hz, 4H), 7.24 (ddt, *J* = 14.3, 12.9, 7.1 Hz, 6H), 6.66 (s, 1H), 6.31 (s, 1H), 4.77 (s, 1H), 3.94 (d, *J* = 14.2 Hz, 1H), 3.89 – 3.77 (m, 4H), 3.70 – 3.58 (m, 4H), 3.39 (s, 3H), 3.10 (dd, *J* = 15.3, 7.4 Hz, 1H), 2.89 (dd, *J* = 15.3, 5.0 Hz, 1H).

^13^C NMR (101 MHz, CDCl_3_) δ 174.02, 147.81, 147.39, 143.22, 138.71, 129.63, 129.14, 129.12, 128.14, 127.98, 127.09, 126.98, 125.94, 111.44, 110.71, 64.79, 60.69, 59.07, 55.93, 55.90, 51.53, 30.55.

Recrystallization from ethyl acetate at room temperature afforded colourless crystals suitable for X-ray analysis.

### Refinement

All non-hydrogen atoms were refined anisotropically. All hydrogen atoms were positioned geometrically with C—H distances ranging from 0.95 Å to 1.00 Å and refined as riding on their parent atoms with *U*_iso_ (H) = 1.2 - 1.5 *U*_eq_ (C). With unmerged reflections the Flack *x* parameter equals to 0.7580 with e.s.d. 0.7487. In the final refinement Friedel pairs were averaged and the *R* factor is 0.0314.

### Crystal data

**Table:**

C_26_H_27_NO_4_	*F*(000) = 444
*M**_r_* = 417.49	*D*_x_ = 1.263 Mg m^−^^3^
Monoclinic, *P*2_1_	Melting point: 420 K
Hall symbol: P 2yb	Mo *K*α radiation, λ = 0.71073 Å
*a* = 9.7797 (7) Å	Cell parameters from 20624 reflections
*b* = 5.4646 (4) Å	θ = 2.0–28.4°
*c* = 20.6959 (15) Å	µ = 0.09 mm^−^^1^
β = 96.986 (1)°	*T* = 173 K
*V* = 1097.82 (14) Å^3^	Needle, yellow
*Z* = 2	0.85 × 0.07 × 0.06 mm

### Data collection

**Table:**

Bruker Kappa DUO APEXII diffractometer	3032 independent reflections
Radiation source: fine-focus sealed tube	2764 reflections with *I* > 2σ(*I*)
graphite	*R*_int_ = 0.030
0.5° φ scans and ω	θ_max_ = 28.4°, θ_min_ = 2.0°
Absorption correction: multi-scan (*SADABS*; Sheldrick, 2008a)	*h* = −13→13
*T*_min_ = 0.931, *T*_max_ = 0.995	*k* = −7→7
20624 measured reflections	*l* = −27→27

### Refinement

**Table:**

Refinement on *F*^2^	Primary atom site location: structure-invariant direct methods
Least-squares matrix: full	Secondary atom site location: difference Fourier map
*R*[*F*^2^ > 2σ(*F*^2^)] = 0.031	Hydrogen site location: inferred from neighbouring sites
*wR*(*F*^2^) = 0.087	H-atom parameters constrained
*S* = 1.05	*w* = 1/[σ^2^(*F*_o_^2^) + (0.0488*P*)^2^ + 0.1593*P*] where *P* = (*F*_o_^2^ + 2*F*_c_^2^)/3
3032 reflections	(Δ/σ)_max_ < 0.001
280 parameters	Δρ_max_ = 0.21 e Å^−^^3^
1 restraint	Δρ_min_ = −0.19 e Å^−^^3^

### Special details

**Table:**

Experimental. Half sphere of data collected using the Bruker *SAINT* software package. Crystal to detector distance = 30 mm; combination of φ and ω scans of 0.5°, 50 s per °, 2 iterations.
Geometry. All e.s.d.'s (except the e.s.d. in the dihedral angle between two l.s. planes) are estimated using the full covariance matrix. The cell e.s.d.'s are taken into account individually in the estimation of e.s.d.'s in distances, angles and torsion angles; correlations between e.s.d.'s in cell parameters are only used when they are defined by crystal symmetry. An approximate (isotropic) treatment of cell e.s.d.'s is used for estimating e.s.d.'s involving l.s. planes.
Refinement. Refinement of *F*^2^ against ALL reflections. The weighted *R*-factor *wR* and goodness of fit *S* are based on *F*^2^, conventional *R*-factors *R* are based on *F*, with *F* set to zero for negative *F*^2^. The threshold expression of *F*^2^ > σ(*F*^2^) is used only for calculating *R*-factors(gt) *etc*. and is not relevant to the choice of reflections for refinement. *R*-factors based on *F*^2^ are statistically about twice as large as those based on *F*, and *R*- factors based on ALL data will be even larger.

### Fractional atomic coordinates and isotropic or equivalent isotropic displacement parameters (Å^2^)

**Table:**

	*x*	*y*	*z*	*U*_iso_*/*U*_eq_	
O1	0.59208 (12)	0.2130 (3)	0.07345 (6)	0.0400 (3)	
O2	0.53131 (12)	−0.1159 (3)	0.15604 (6)	0.0370 (3)	
O3	1.13983 (13)	0.2008 (3)	0.41963 (6)	0.0382 (3)	
O4	1.06894 (12)	0.5827 (3)	0.43713 (6)	0.0371 (3)	
N1	1.07939 (12)	0.4482 (3)	0.28290 (6)	0.0250 (3)	
C1	0.99153 (14)	0.5106 (3)	0.22148 (7)	0.0248 (3)	
H1	0.9572	0.6819	0.2252	0.030*	
C2	0.86750 (14)	0.3416 (3)	0.20811 (7)	0.0241 (3)	
C3	0.78789 (15)	0.3561 (3)	0.14696 (7)	0.0290 (3)	
H3	0.8117	0.4713	0.1158	0.035*	
C4	0.67576 (15)	0.2054 (4)	0.13156 (7)	0.0290 (3)	
C5	0.64159 (15)	0.0305 (3)	0.17670 (7)	0.0278 (3)	
C6	0.71774 (15)	0.0206 (3)	0.23736 (7)	0.0261 (3)	
H6	0.6943	−0.0953	0.2684	0.031*	
C7	0.82981 (14)	0.1801 (3)	0.25388 (7)	0.0238 (3)	
C8	0.90596 (15)	0.1782 (3)	0.32152 (7)	0.0262 (3)	
H8A	0.9673	0.0336	0.3267	0.031*	
H8B	0.8390	0.1647	0.3536	0.031*	
C9	0.99109 (14)	0.4091 (3)	0.33493 (7)	0.0246 (3)	
H9	0.9278	0.5523	0.3364	0.030*	
C10	1.07737 (14)	0.3815 (4)	0.40099 (7)	0.0279 (3)	
C11	1.1481 (2)	0.5762 (5)	0.50083 (9)	0.0495 (5)	
H11A	1.1357	0.7303	0.5236	0.074*	
H11B	1.2459	0.5541	0.4961	0.074*	
H11C	1.1165	0.4397	0.5259	0.074*	
C12	1.17749 (15)	0.6521 (3)	0.29833 (8)	0.0291 (3)	
H12A	1.1310	0.7808	0.3213	0.035*	
H12B	1.1998	0.7234	0.2569	0.035*	
C13	1.31095 (14)	0.5860 (3)	0.33944 (7)	0.0270 (3)	
C14	1.38533 (17)	0.3781 (4)	0.32668 (9)	0.0355 (4)	
H14	1.3478	0.2647	0.2945	0.043*	
C15	1.51460 (19)	0.3358 (4)	0.36092 (11)	0.0469 (5)	
H15	1.5656	0.1948	0.3516	0.056*	
C16	1.56899 (19)	0.4983 (5)	0.40837 (11)	0.0501 (5)	
H16	1.6573	0.4691	0.4317	0.060*	
C17	1.4954 (2)	0.7017 (5)	0.42181 (10)	0.0482 (5)	
H17	1.5325	0.8125	0.4547	0.058*	
C18	1.36621 (17)	0.7465 (4)	0.38741 (9)	0.0369 (4)	
H18	1.3158	0.8879	0.3969	0.044*	
C19	1.07526 (15)	0.4993 (3)	0.16403 (8)	0.0280 (3)	
C20	1.16294 (16)	0.3043 (3)	0.15664 (8)	0.0322 (4)	
H20	1.1761	0.1814	0.1893	0.039*	
C21	1.23198 (19)	0.2875 (4)	0.10159 (9)	0.0409 (4)	
H21	1.2937	0.1558	0.0973	0.049*	
C22	1.2103 (2)	0.4635 (5)	0.05320 (10)	0.0481 (5)	
H22	1.2556	0.4504	0.0152	0.058*	
C23	1.1231 (2)	0.6574 (5)	0.06006 (10)	0.0489 (5)	
H23	1.1079	0.7772	0.0267	0.059*	
C24	1.05718 (18)	0.6783 (4)	0.11589 (9)	0.0380 (4)	
H24	0.9997	0.8153	0.1211	0.046*	
C25	0.60475 (19)	0.4241 (4)	0.03433 (8)	0.0400 (4)	
H25A	0.5409	0.4110	−0.0059	0.060*	
H25B	0.6994	0.4361	0.0236	0.060*	
H25C	0.5827	0.5705	0.0584	0.060*	
C26	0.4896 (2)	−0.2845 (4)	0.20218 (9)	0.0442 (5)	
H26A	0.4106	−0.3792	0.1821	0.066*	
H26B	0.4636	−0.1948	0.2398	0.066*	
H26C	0.5660	−0.3957	0.2164	0.066*	

### Atomic displacement parameters (Å^2^)

**Table:**

	*U*^11^	*U*^22^	*U*^33^	*U*^12^	*U*^13^	*U*^23^
O1	0.0368 (6)	0.0521 (9)	0.0288 (5)	−0.0133 (7)	−0.0052 (4)	0.0025 (6)
O2	0.0323 (6)	0.0430 (8)	0.0351 (6)	−0.0161 (6)	0.0015 (5)	−0.0048 (6)
O3	0.0354 (6)	0.0417 (8)	0.0358 (6)	0.0060 (6)	−0.0023 (5)	0.0019 (6)
O4	0.0308 (6)	0.0452 (8)	0.0345 (6)	0.0012 (6)	0.0007 (5)	−0.0165 (6)
N1	0.0212 (5)	0.0238 (7)	0.0298 (6)	−0.0033 (5)	0.0022 (4)	−0.0016 (5)
C1	0.0225 (6)	0.0217 (7)	0.0303 (7)	−0.0023 (6)	0.0035 (5)	−0.0001 (6)
C2	0.0204 (6)	0.0245 (8)	0.0276 (7)	−0.0022 (6)	0.0039 (5)	−0.0023 (6)
C3	0.0267 (6)	0.0321 (9)	0.0283 (7)	−0.0059 (7)	0.0041 (5)	0.0028 (7)
C4	0.0247 (6)	0.0364 (9)	0.0257 (7)	−0.0032 (7)	0.0018 (5)	−0.0022 (7)
C5	0.0232 (6)	0.0307 (9)	0.0301 (7)	−0.0066 (6)	0.0059 (5)	−0.0064 (7)
C6	0.0251 (6)	0.0262 (8)	0.0281 (7)	−0.0040 (6)	0.0072 (5)	−0.0010 (6)
C7	0.0213 (6)	0.0250 (7)	0.0257 (6)	−0.0006 (6)	0.0046 (5)	−0.0027 (6)
C8	0.0241 (6)	0.0280 (8)	0.0265 (6)	−0.0014 (6)	0.0030 (5)	0.0002 (7)
C9	0.0211 (6)	0.0251 (8)	0.0272 (6)	0.0028 (6)	0.0012 (5)	−0.0044 (6)
C10	0.0211 (6)	0.0339 (9)	0.0291 (7)	−0.0001 (6)	0.0046 (5)	−0.0046 (7)
C11	0.0402 (9)	0.0716 (16)	0.0348 (9)	−0.0054 (11)	−0.0031 (7)	−0.0192 (11)
C12	0.0232 (7)	0.0226 (8)	0.0403 (8)	−0.0013 (6)	−0.0003 (6)	−0.0016 (7)
C13	0.0211 (6)	0.0266 (8)	0.0338 (7)	−0.0012 (6)	0.0054 (5)	0.0013 (7)
C14	0.0328 (8)	0.0311 (9)	0.0430 (9)	0.0040 (7)	0.0056 (7)	−0.0025 (8)
C15	0.0338 (9)	0.0422 (12)	0.0649 (13)	0.0124 (9)	0.0070 (8)	0.0056 (10)
C16	0.0280 (8)	0.0554 (13)	0.0630 (12)	0.0030 (9)	−0.0095 (8)	0.0129 (11)
C17	0.0363 (9)	0.0501 (13)	0.0539 (11)	−0.0081 (10)	−0.0119 (8)	−0.0049 (11)
C18	0.0279 (7)	0.0333 (10)	0.0481 (9)	−0.0010 (7)	−0.0003 (7)	−0.0072 (8)
C19	0.0240 (7)	0.0270 (8)	0.0332 (7)	−0.0086 (6)	0.0047 (5)	−0.0008 (7)
C20	0.0303 (7)	0.0307 (9)	0.0360 (8)	−0.0057 (7)	0.0059 (6)	−0.0047 (7)
C21	0.0372 (9)	0.0410 (11)	0.0464 (10)	−0.0078 (8)	0.0129 (7)	−0.0133 (9)
C22	0.0468 (10)	0.0592 (14)	0.0421 (9)	−0.0157 (11)	0.0204 (8)	−0.0061 (10)
C23	0.0505 (11)	0.0531 (14)	0.0451 (10)	−0.0139 (10)	0.0135 (8)	0.0131 (10)
C24	0.0352 (8)	0.0349 (10)	0.0448 (9)	−0.0070 (8)	0.0085 (7)	0.0067 (8)
C25	0.0412 (9)	0.0465 (11)	0.0308 (8)	0.0035 (9)	−0.0019 (7)	0.0022 (9)
C26	0.0437 (9)	0.0465 (12)	0.0438 (9)	−0.0241 (10)	0.0108 (7)	−0.0077 (10)

### Geometric parameters (Å, °)

**Table:**

O1—C4	1.3705 (17)	C12—H12A	0.9900
O1—C25	1.423 (3)	C12—H12B	0.9900
O2—C5	1.3688 (18)	C13—C18	1.384 (2)
O2—C26	1.422 (2)	C13—C14	1.391 (2)
O3—C10	1.200 (2)	C14—C15	1.391 (3)
O4—C10	1.338 (2)	C14—H14	0.9500
O4—C11	1.446 (2)	C15—C16	1.382 (3)
N1—C9	1.4755 (18)	C15—H15	0.9500
N1—C12	1.480 (2)	C16—C17	1.371 (3)
N1—C1	1.4849 (18)	C16—H16	0.9500
C1—C2	1.523 (2)	C17—C18	1.394 (2)
C1—C19	1.525 (2)	C17—H17	0.9500
C1—H1	1.0000	C18—H18	0.9500
C2—C7	1.378 (2)	C19—C20	1.388 (2)
C2—C3	1.405 (2)	C19—C24	1.392 (3)
C3—C4	1.377 (2)	C20—C21	1.396 (2)
C3—H3	0.9500	C20—H20	0.9500
C4—C5	1.405 (2)	C21—C22	1.386 (3)
C5—C6	1.381 (2)	C21—H21	0.9500
C6—C7	1.409 (2)	C22—C23	1.378 (4)
C6—H6	0.9500	C22—H22	0.9500
C7—C8	1.5035 (19)	C23—C24	1.395 (3)
C8—C9	1.519 (2)	C23—H23	0.9500
C8—H8A	0.9900	C24—H24	0.9500
C8—H8B	0.9900	C25—H25A	0.9800
C9—C10	1.524 (2)	C25—H25B	0.9800
C9—H9	1.0000	C25—H25C	0.9800
C11—H11A	0.9800	C26—H26A	0.9800
C11—H11B	0.9800	C26—H26B	0.9800
C11—H11C	0.9800	C26—H26C	0.9800
C12—C13	1.513 (2)		
			
C4—O1—C25	116.09 (14)	C13—C12—H12A	108.4
C5—O2—C26	116.63 (13)	N1—C12—H12B	108.4
C10—O4—C11	115.28 (16)	C13—C12—H12B	108.4
C9—N1—C12	111.91 (12)	H12A—C12—H12B	107.4
C9—N1—C1	109.20 (11)	C18—C13—C14	119.07 (15)
C12—N1—C1	107.71 (12)	C18—C13—C12	119.12 (16)
N1—C1—C2	112.42 (12)	C14—C13—C12	121.58 (15)
N1—C1—C19	110.39 (11)	C15—C14—C13	120.17 (18)
C2—C1—C19	108.97 (12)	C15—C14—H14	119.9
N1—C1—H1	108.3	C13—C14—H14	119.9
C2—C1—H1	108.3	C16—C15—C14	120.2 (2)
C19—C1—H1	108.3	C16—C15—H15	119.9
C7—C2—C3	119.35 (13)	C14—C15—H15	119.9
C7—C2—C1	122.51 (13)	C17—C16—C15	119.92 (17)
C3—C2—C1	118.13 (13)	C17—C16—H16	120.0
C4—C3—C2	120.88 (15)	C15—C16—H16	120.0
C4—C3—H3	119.6	C16—C17—C18	120.29 (19)
C2—C3—H3	119.6	C16—C17—H17	119.9
O1—C4—C3	123.96 (15)	C18—C17—H17	119.9
O1—C4—C5	116.04 (14)	C13—C18—C17	120.35 (19)
C3—C4—C5	119.99 (13)	C13—C18—H18	119.8
O2—C5—C6	125.36 (15)	C17—C18—H18	119.8
O2—C5—C4	115.59 (13)	C20—C19—C24	119.15 (15)
C6—C5—C4	119.04 (14)	C20—C19—C1	121.00 (15)
C5—C6—C7	120.93 (14)	C24—C19—C1	119.72 (16)
C5—C6—H6	119.5	C19—C20—C21	120.48 (18)
C7—C6—H6	119.5	C19—C20—H20	119.8
C2—C7—C6	119.65 (13)	C21—C20—H20	119.8
C2—C7—C8	120.07 (13)	C22—C21—C20	119.81 (19)
C6—C7—C8	120.27 (13)	C22—C21—H21	120.1
C7—C8—C9	111.28 (13)	C20—C21—H21	120.1
C7—C8—H8A	109.4	C23—C22—C21	120.08 (17)
C9—C8—H8A	109.4	C23—C22—H22	120.0
C7—C8—H8B	109.4	C21—C22—H22	120.0
C9—C8—H8B	109.4	C22—C23—C24	120.2 (2)
H8A—C8—H8B	108.0	C22—C23—H23	119.9
N1—C9—C8	110.08 (12)	C24—C23—H23	119.9
N1—C9—C10	111.08 (11)	C19—C24—C23	120.3 (2)
C8—C9—C10	108.24 (14)	C19—C24—H24	119.9
N1—C9—H9	109.1	C23—C24—H24	119.9
C8—C9—H9	109.1	O1—C25—H25A	109.5
C10—C9—H9	109.1	O1—C25—H25B	109.5
O3—C10—O4	124.06 (14)	H25A—C25—H25B	109.5
O3—C10—C9	125.08 (16)	O1—C25—H25C	109.5
O4—C10—C9	110.79 (14)	H25A—C25—H25C	109.5
O4—C11—H11A	109.5	H25B—C25—H25C	109.5
O4—C11—H11B	109.5	O2—C26—H26A	109.5
H11A—C11—H11B	109.5	O2—C26—H26B	109.5
O4—C11—H11C	109.5	H26A—C26—H26B	109.5
H11A—C11—H11C	109.5	O2—C26—H26C	109.5
H11B—C11—H11C	109.5	H26A—C26—H26C	109.5
N1—C12—C13	115.70 (14)	H26B—C26—H26C	109.5
N1—C12—H12A	108.4		
			
C9—N1—C1—C2	−46.18 (17)	C1—N1—C9—C10	−171.68 (14)
C12—N1—C1—C2	−167.91 (12)	C7—C8—C9—N1	−52.11 (16)
C9—N1—C1—C19	−168.08 (13)	C7—C8—C9—C10	−173.69 (12)
C12—N1—C1—C19	70.19 (16)	C11—O4—C10—O3	3.4 (2)
N1—C1—C2—C7	10.8 (2)	C11—O4—C10—C9	−179.57 (14)
C19—C1—C2—C7	133.55 (15)	N1—C9—C10—O3	−76.9 (2)
N1—C1—C2—C3	−170.35 (13)	C8—C9—C10—O3	44.1 (2)
C19—C1—C2—C3	−47.65 (19)	N1—C9—C10—O4	106.16 (15)
C7—C2—C3—C4	−2.2 (2)	C8—C9—C10—O4	−132.88 (14)
C1—C2—C3—C4	178.97 (15)	C9—N1—C12—C13	85.52 (16)
C25—O1—C4—C3	−14.8 (2)	C1—N1—C12—C13	−154.45 (13)
C25—O1—C4—C5	165.37 (15)	N1—C12—C13—C18	−140.47 (16)
C2—C3—C4—O1	178.63 (16)	N1—C12—C13—C14	45.0 (2)
C2—C3—C4—C5	−1.5 (3)	C18—C13—C14—C15	−1.3 (3)
C26—O2—C5—C6	2.6 (2)	C12—C13—C14—C15	173.24 (17)
C26—O2—C5—C4	−176.36 (16)	C13—C14—C15—C16	0.9 (3)
O1—C4—C5—O2	2.0 (2)	C14—C15—C16—C17	0.1 (3)
C3—C4—C5—O2	−177.87 (15)	C15—C16—C17—C18	−0.5 (3)
O1—C4—C5—C6	−177.02 (15)	C14—C13—C18—C17	0.8 (3)
C3—C4—C5—C6	3.1 (2)	C12—C13—C18—C17	−173.86 (17)
O2—C5—C6—C7	180.00 (15)	C16—C17—C18—C13	0.1 (3)
C4—C5—C6—C7	−1.1 (2)	N1—C1—C19—C20	45.6 (2)
C3—C2—C7—C6	4.2 (2)	C2—C1—C19—C20	−78.34 (17)
C1—C2—C7—C6	−177.02 (14)	N1—C1—C19—C24	−138.67 (15)
C3—C2—C7—C8	−174.44 (14)	C2—C1—C19—C24	97.42 (18)
C1—C2—C7—C8	4.3 (2)	C24—C19—C20—C21	−0.1 (2)
C5—C6—C7—C2	−2.6 (2)	C1—C19—C20—C21	175.66 (15)
C5—C6—C7—C8	176.03 (14)	C19—C20—C21—C22	−1.6 (3)
C2—C7—C8—C9	16.00 (19)	C20—C21—C22—C23	1.4 (3)
C6—C7—C8—C9	−162.63 (14)	C21—C22—C23—C24	0.5 (3)
C12—N1—C9—C8	−172.39 (12)	C20—C19—C24—C23	2.0 (3)
C1—N1—C9—C8	68.45 (16)	C1—C19—C24—C23	−173.83 (16)
C12—N1—C9—C10	−52.52 (18)	C22—C23—C24—C19	−2.2 (3)

### Hydrogen-bond geometry (Å, °)

**Table:**

*D*—H···*A*	*D*—H	H···*A*	*D*···*A*	*D*—H···*A*
C18—H18···O3^i^	0.95	2.51	3.445 (2)	168
C25—H25A···O1^ii^	0.98	2.43	3.183 (2)	133

Symmetry codes: (i) *x*, *y*+1, *z*; (ii) −*x*+1, *y*+1/2, −*z*.
